# Predictive Validity of Motor Assessment Scale on Poststroke Discharge Destination

**DOI:** 10.1155/2024/2914252

**Published:** 2024-07-20

**Authors:** Irene Conradsen, Marius Henriksen, Hana Malá Rytter

**Affiliations:** ^1^ Department of Physical and Occupational Therapy Copenhagen University Hospital Bispebjerg-Frederiksberg, Copenhagen, Denmark; ^2^ Center for Rehabilitation of Brain Injury, Copenhagen, Denmark; ^3^ The Parker Institute Copenhagen University Hospital Bispebjerg-Frederiksberg, Copenhagen, Denmark; ^4^ Department of Clinical Medicine Faculty of Health and Medical Sciences University of Copenhagen, Copenhagen, Denmark; ^5^ Department of Neurology Copenhagen University Hospital Bispebjerg-Frederiksberg, Copenhagen, Denmark; ^6^ Department of Psychology University of Copenhagen, Copenhagen, Denmark

**Keywords:** MAS, Motor Assessment Scale, motor function, rehabilitation, stroke, validity

## Abstract

**Background:** Stroke frequently leads to hospital admission and subsequent rehabilitation in order to overcome poststroke sequelae, such as motor impairments. Efficient planning of the steps following hospital admission includes early prediction of whether the patient can be discharged home or not. Early assessment of motor performance in patients with stroke-induced motor deficits may be able to function as a predictor of discharge destination but is less explored.

**Objective:** The primary objective was to assess the predictive validity of the Motor Assessment Scale (MAS) on discharge destination both regarding total score and regarding subscores (transfer-mobility items and upper extremity items).

**Design:** The study was designed as a prospective cohort study.

**Subjects:** Thirty-seven consecutively recruited patients with stroke are the subjects of the study.

**Methods:** Logistic regression model was used to calculate the odds of being discharged to own home upon hospital admittance. The predictive ability was examined with a receiving operator characteristic (ROC) curve, and cut-points from the curve were employed in Cox regression.

**Results:** A one-unit higher score on the total MAS significantly increased the odds of being discharged home upon hospital admittance (odds ratio (OR) 1.14, 95% CI 1.04–1.25). The same pattern was observed with the summed items of 1–5 and 6–8. The total MAS showed sensitivity of 91.7% and specificity of 68.0%. Patients having a total MAS score ≥ 24 were 17 times more likely to be discharged home (HR 17.64, 95% CI 2.23–139.57) compared to patients with a lower score.

**Conclusion:** Motor function measured by the MAS can be applied as a predictor of discharge destination upon hospital admission after stroke in Danish setting.

## 1. Introduction

Cerebrovascular stroke is responsible for approximately 6.5 million deaths per year and 143 million disability-adjusted life year (DALY) worldwide [1, 2]. Annually in Denmark (population of approx. 5.9 million), there is approximately 12,000 persons who experience stroke while 110,000 persons live with the diagnosis of stroke [3, 4]. In 2010, the DALY for stroke totalled 67,627 in Denmark [3]. Motor function is affected in the majority of stroke survivors [5].

Early supported discharge has been shown to be effective in reducing long-term disability following stroke [6]. Stroke patients are admitted to acute neurological wards and, if necessary, transferred to early subacute stroke wards. In Denmark, patients transferred to subacute stroke wards have an expected average length of hospital stay of approximately 16–18 days [7]. After hospital discharge, municipalities assume the responsibilities for providing rehabilitation services. If patients are unable to be discharged home when medically stable, they are transferred to municipal rehabilitation clinics [8]. An efficient collaboration and planning between hospitals and municipal rehabilitation clinics includes the possibility of making an early distinction between patients who can be discharged to their home and patients who will be discharged to municipal rehabilitation clinics.

Parameters known to predict poststroke discharge destination are demographic factors (age, cohabitation status, prestroke housing situation, alcohol abuse, and comorbidities), stroke characteristics (stroke severity, physical functioning poststroke, cognitive status, and severe dysphagia), and a body mass index (BMI) above 35 [9, 10]. Data on the association between sex, the type of stroke (ischemic or haemorrhagic), the affected brain hemisphere, and discharge destination are less clear. Additionally, it is widely known that stroke severity and independency scales, such as the Barthel Index (BI) and Functional Independence Measure (FIM), can predict discharge destination [9, 10]. Measuring motor function by using specific instruments to predict discharge destination after a stroke has only been examined by a few studies worldwide [9, 10], and to our knowledge, no study has addressed this issue in a Danish context.

Measurement of physical functioning in patients with stroke covers a broad range of test. Scales aiming to measure motor recovery are, for example, the Fugl-Meyer Motor Assessment (FMA) [11, 12] (at impairment level) and the Motor Assessment Scale (MAS) [13] (at functional level). Both scales are aimed at comparing movement patterns of the affected individual with the natural movement pattern of nonaffected individuals. Contrary to these, measures of independence such as the BI [14] and FIM [15] do not evaluate movement quality or involvement of paretic limb in activities.

The MAS is a unidimensional test measuring motor performance in patients with stroke developed by Carr and Shepherd in 1985 [13]. MAS was implemented in the subacute neurological rehabilitation ward at Bispebjerg-Frederiksberg University Hospital, Denmark, in 2014 with the intention to provide an evidence-based assessment of motor function in patients with stroke and to provide information on hospital discharge. Two international studies have found the MAS to be a valid predictor of discharge destination [16, 17]; however, it is unknown whether these results apply to Danish settings. The MAS has been translated into Danish and validated against FMA [18] but has not been studied whether the Danish translation of the MAS can be employed as a valid predictive tool of discharge destination within the Danish settings with a short admittance time.

The objective of the study was to evaluate whether motor function, as measured by the MAS, can serve as a predictor of discharge destination in medically stable poststroke patients without delirium or severe cognitive disabilities. We therefore assessed the predictive ability of the MAS on discharge destination. We aimed to examine both the predictive validity of the total summed score, of the summed transfer-mobility items of MAS (Items 1–5), and of the summed upper extremity items of MAS (Items 6–8) on discharge destination. Additionally, we aimed to identify optimal cut-points of the MAS regarding discharge destination (discharged home or not), dependent on whether the patient scored below or above the cut-point. We hypothesized that a higher MAS score would be associated with higher odds for discharge home. We also hypothesized that the summed score of the total MAS would be moderately correlated (rho > 30) with discharge destination since other factors like age, cohabitation status, prestroke housing situation, alcohol abuse, comorbidities, medical or surgical treatment, cognitive status, and severe dysphagia were expected to affect the association. Further, we hypothesized that the summed items of MAS subscales transfer-mobility and upper extremity, respectively, would show a similar direction of association with discharge destination as the total MAS, although the association was expected to be weaker in the upper extremity items due to nonhierarchical structure of Items 7 and 8 [19–21]. We hypothesized that patients scoring above the identified cut-points would have higher chances of being discharged home.

## 2. Materials and Methods

### 2.1. Design

The study was designed as a prospective cohort study.

### 2.2. Setting

The study took place at a subacute stroke unit offering early interdisciplinary inpatient rehabilitation at Bispebjerg-Frederiksberg Hospital, Capital Region of Denmark. Patients with motor impairments were referred to physiotherapeutic rehabilitation.

### 2.3. Participants

Participants were patients admitted to the stroke rehabilitation unit from 1st February 2017 to 30th April 2017. They were diagnosed with intracerebral haemorrhage (I61) or cerebral infarction (I63) according to the ICD-10 based on their clinical signs and neuroradiological imaging findings. Patients referred to physiotherapeutic rehabilitation were considered eligible if they complied with following inclusion criteria: (i) age > 18 years and (ii) able to give informed consent. The examination by the physiotherapist was typically carried out after the 1st week of hospitalization. The hospitalization lasted on average 18 days (see Table 1). Patients isolated due to a contagious infection, who were medically unstable (i.e., suffering from poststroke medical complications such as infections, recurrent stroke, and epileptic seizures), had delirium, or who were unable to participate in the testing due to, for example, cognitive disabilities, were excluded. All information was collected from the patients' hospital records.

### 2.4. Measurements

#### 2.4.1. MAS

MAS measures the progress in motor function in patients with stroke with a scale consisting of 8 items [13]. Every item is scored on a 7-point ordinal scale ranging from 0 to 6 based on quality and hierarchic difficulty of each task. Higher score indicates higher level of motor function. MAS is a unidimensional scale that is typically used as individual items. However, the individual items can be summed up to give either a total score (total MAS, range 0–48) or separate partial scores for transfer-mobility Items 1–5 (transfer-mobility MAS, range 0–30) and upper extremity Items 6–8 (UE MAS, range 0–18) [19, 21, 22]. MAS has good psychometric properties with regard to reliability [13, 17, 23, 24] and validity [13, 24], except problems with the hierarchical order of Item 8 [20, 21, 25]. The Danish translation of MAS [18, 26] consists of eight items with six tasks in each item. In this study, MAS was applied within three weekdays from admittance to the stroke rehabilitation unit. All physiotherapists were introduced to and calibrated to the MAS per protocol [27] to assure the reliability of the test.

#### 2.4.2. Discharge Destination

Information on discharge destination was collected from the medical record at discharge and divided into five categories: (i) discharged home, (ii) discharged to a municipal rehabilitation unit, (iii) discharged to another hospital unit, (iv) discharged to a nursing home, and (v) death during admittance.

#### 2.4.3. Patient and Injury Characteristics

Information about prestroke residential status (home, rehabilitation unit, or nursing home), cohabitant status (alone/cohabitant), motor sequelae from previous stroke (yes/no), pulse, blood pressure and BMI, affected cerebral hemisphere (right, left, and bilateral), and stroke type (ischemic/haemorrhagic) was collected from the hospital records. Treatment with thrombolysis, endovascular thrombectomy (EVT), or craniectomy was registered (additional treatment yes/no). Dysphagia was evaluated with the Functional Oral Intake Scale (FOIS) [28] by an occupational therapist [29]. FOIS ranks the patient's capability of functional oral intake into seven levels, Level 1 indicating inability to receive any food or beverage through the mouth and Level 7 indicating eating and drinking without restrictions [28]. The scale has shown good interrater reliability and validity in patients with stroke [28, 30].

### 2.5. Statistical Analyses

Distribution of data on the MAS test and discharge destination were explored prior to the statistical analyses to reveal potential weakness in the analyses (e.g., skewness or clustering of data). Descriptive statistics was performed using mean and standard deviation (SD) when data were continuous variables, by median and interquartile range (IQR) when data were ordinal variables, and by frequency and percentage when data were nominal and binary variables. Logistic regression was applied to calculate odds ratios (ORs) between MAS score and discharge destination. Since interaction analysis revealed a tendency toward interaction between age and MAS score, this interaction was included into the logistic regression analysis (age by MAS). Discharge destination was dichotomized into being discharged home or not. In case of missing data regarding discharge destination, the case was excluded from the regression analyses. Cut-points of the MAS score, predicting whether a patient would be discharged home, were determined with a receiving operator characteristic (ROC) curve analysis, with regard to sensitivity and specificity. The study population was dichotomized according to the selected cut-point, and the association between group and discharge destination across time was analyzed with the Kaplan–Meier estimate of survival curve and Cox regression. A sensitivity analysis of Cox regression, applying different cut-points of the MAS score, was employed to evaluate whether optimal cut-points had been chosen.

### 2.6. Ethical Approval

The study was approved by the Danish Data Protection Agency, journal number 05307 and BFH-2017-012, and by the National Committee on Health Research Ethics, Capital Region of Denmark, journal number 17002843. All participants signed a written consent.

## 3. Results and Discussion

During the inclusion period, 88 patients were referred to physiotherapeutic rehabilitation after stroke at the stroke rehabilitation unit. Twenty-nine of these were unable to give informed consent due to dementia, severe aphasia, or cognitive disability poststroke, and three patients declined to participate. Eight patients were not tested with the MAS due to isolation, administrative reasons, or declined to perform the test. Eleven patients were unable to perform the MAS due to cognitive disability, somnolence, or an unstable medical condition. Thirty-seven patients were able to perform the MAS and were included in the study. There were no dropouts and no missing data except for 2.7% missing data on BMI. Patient and injury characteristics are summarized in Table 1. Outcome measures are presented in Table 2.

There was a moderate correlation (Rho 0.536, *p* < 0.001) between the total MAS and discharge destination, with none of the patients with a score below 19 points being discharged home after hospital admittance. A similar pattern was observed for the transfer-mobility MAS (Rho 0.501, *p* = 0.002) and the UE MAS (Rho 0.535, *p* = 0.001).

The binary logistic regression analysis between the total MAS and discharge destination showed that a higher score at admittance significantly increased the odds of being discharged home after hospital admittance compared to a lower score (OR 1.14, 95% CI 1.04–1.25). The variance in the total MAS explained 44.3% of the variance in discharge destination. Adjusting for potential confounders and predictors such as age, additional treatment, prestroke residential status, and cohabitant status yielded a higher OR of being discharged home with a higher score (OR 1.30, 95% CI 1.02–1.65). The variance in the variables included in the multiple logistic regression analysis explained 48.2% of the variance in discharge destination (see Table 3).

A similar pattern was observed for the transfer-mobility MAS subscale (OR 1.17, 95% CI 1.04–1.32) and the UE MAS subscale (OR 1.28, 95% CI 1.08–1.52) with higher score increasing the odds of being discharged home. When adjusted for potential confounders and predictors, the OR increased, although the effect of the UE MAS subscale was nonsignificant.

**Figure 1:**
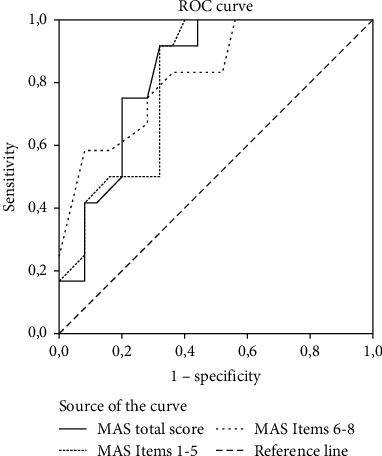
The ROC curve for total MAS, transfer-ability MAS, and UE MAS. *X*-axis shows specificity as the ratio between the persons predicted not to be discharged home and all the persons who were not discharged (1-specificity). *Y*-axis shows sensitivity as the ratio between the persons who were predicted to be discharged home and all the persons who were discharged home (sensitivity). Interrupted diagonal line: reference line. Full black line: the MAS total score. Dotted black line: the partial score for Items 1–5, transfer-mobility MAS. Spaced dotted black line: the partial score for Items 6–8, upper extremity MAS.

Results from the ROC curve analysis indicated a significant and good predictive ability of the total MAS (area under curve (AUC) 0.83, 95% CI 0.70–0.96), the transfer-mobility MAS (AUC 0.81, 95% CI 0.67–0.95), and the UE MAS (AUC 0.83, 95% CI 0.69–0.97) on discharge destination (see Figure 1).

Employing the coordinates of the ROC curve, optimal cut-points for the MAS were chosen as a trade-off between sensitivity and specificity. A cut-point of primarily high sensitivity was chosen since this would help identify a higher amount of true positive cases and permit planning of discharge to own home within the first week of admittance. At the same time, a very low specificity could not be tolerated since an increase in the number of false positives would be costly to the hospital departments and yield unrealistic goal setting with the patients. In the total MAS, a cut-point of ≥ 24 showed a sensitivity of 91.7% and a specificity of 68.0%, yielding an overall predictive value 75.7%.

In the transfer-mobility MAS, the cut-point was set at 15.5 with a sensitivity of 91.7% and a specificity of 68.00%. Although a cut-point of 13 yielded the lowest amount of false results, this was mainly caused by 100.0% sensitivity and a lower specificity of 60.0%. Hence, a cut-point of 15.5 was chosen instead. In the UE MAS, the cut-point of 6.5 yielded the highest sensitivity of 83.3% and specificity of 64%.

The cut-points from the ROC curve were employed to create two groups, a group with a low MAS score and a group with a high score. In the total MAS, the probability that an individual having a high score (≥ 24 points) was experiencing the event of being discharged home after hospital admittance was 17 times more likely compared to a patient with a low score (≤ 23 points) (HR 17.64, 95% CI 2.23–139.57). The same pattern was observed for the transfer-mobility MAS with a cut-point of > 15 points (HR 17.02, 95% CI 2.15–135.01) and for the UE MAS with a cut-point of > 6 points (HR 7.11, 95% CI 1.54–32.83) (see Table 3). The sensitivity analyses of Cox regression indicated that the optimal cut-points had been chosen. Data are not shown.

**Figure 2:**
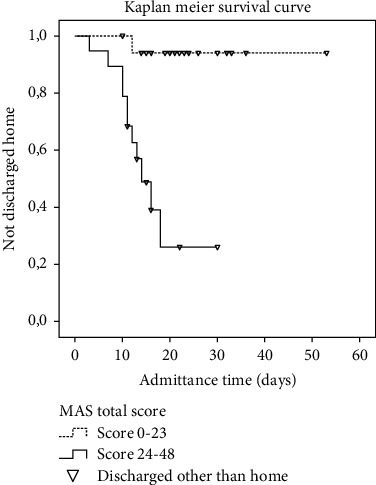
The Kaplan–Meier survival curve showing the discharge destination in patients with high versus low total MAS score. *X*-axis shows the length of hospital admittance, the *Y*-axis percentage of patients not discharged home. The black dotted line: patients with total MAS score ≤ 23. The black solid line: patients with total MAS score ≥ 24. Downward pointing triangle: subject discharged to destinations other than home.

The difference in discharge destination between the two groups having a high and a low MAS score was visualised with the Kaplan–Meier survival curve. A significant difference in the event of being discharged home after hospital admittance was found in the total MAS, the transfer-mobility MAS, and the UE MAS. In the total MAS, approximately 58% of the patients with a MAS score ≥ 24 were discharged to their own home after 18 days of hospital admittance, whereas only approximately 6% of patients with a score of ≤ 23 were discharged home at that point of time. Patients with a higher MAS scores had a shorter admittance time compared to patients with a low score. None of the patients being admitted for more than 18 days was discharged directly home (see Figure 2).

On the individual item level, Item 4 (sitting to standing) and Item 8 (advanced hand activities) were the strongest and most solid predictors of being discharged home poststroke in binary logistic analysis and ROC curve. Item 4 explained that 38.5% of the variance in discharge destination and a cut-point of 2, equal to being able to stand-up with stand-by help, was indicated in the ROC curve as differentiating between being discharged home or not. Likewise, Item 8 explained that 33.7% of the variance in discharge destination and a cut-off point of 1, equal to being able to pick up the top of a pen and putting it down again with affected hand, was indicated in the ROC curve as differentiating between being discharged home or not (see Table 4).

## 4. Discussion

This prospective cohort study evaluated the predictive validity of the MAS on poststroke discharge destination. The results show that the higher the MAS score, the higher are the odds of being discharged home (OR 1.14, 95% CI 1.04–1.25). Comparing the predictive validity of the total MAS score (Items 1–8), the transfer-mobility subscale (Items 1–5) and UE subscale (Items 6–8) did not yield different results. Thus, both the total MAS and the subscales can serve as predictive tools regarding discharge destination.

The ROC curve analysis showed a good predictive ability with an AUC of 0.83 (95% CI 0.70–0.96). A cut-point of ≥ 24 in the total MAS from the ROC curve showed a sensitivity of 91.7% and specificity of 68.0%. Dividing the study population according to this cut-point, the group having a high total MAS score was 17 times more likely to be discharged home compared to the group having a low score (HR 17.64, 95% CI 2.23–139.57).

Brauer et al. [16] examined the predictability of the MAS on poststroke discharge destination in 502 patients and found that a one-unit increase in Item 5 and Item 1 increased the OR of being discharged home by 1.66 (95% CI 1.28–2.27) and 1.28 (95% CI 1.11–1.49), respectively. This is the same direction and somewhat similar effect size as in the current study (OR 1.39, 95% CI 0.99–1.96 in Item 5 and OR 2.07 95%, CI 1.02–4.19 in Item 1), though the confidence intervals in this study were wider, presumably due to a smaller sample size. Brauer et al. found a sensitivity of 99% and a specificity of 33% predicting discharge destination based on a predictive model of MAS Items 1 and 5, prestroke living situation and age. In our study, a similar sensitivity of 91.7% was found, though a higher specificity of 68.0% was identified. Had we chosen a sensitivity of 100%, the specificity would have been 60% compared to 33% found by Brauer et al. This suggests that a total summed score might increase the specificity. Brauer et al. found an overall predictive value of 87%, which is higher than the 75.7% in our study. One explanation for this lower overall predictive value may be due to a lower number of patients discharged home in our study (32%) compared to the 81% in the Brauer et al. study [16].

Tucak et al. [17] applied the MAS as a predictor of discharge destination in a population with a predefined rehabilitation potential aged > 65 years (*n* = 239). They found that a model including the total MAS score (subdivided into four categories on admission) and age predicted discharge destination with a sensitivity of 87% and a specificity of 51%. This is a bit lower than the sensitivity and specificity of our study. It is possible that testing motor functioning with the MAS early poststroke (mean 6 days) as in our study yielded a stronger predictor than when the testing takes place later as seen in the study by Tucak et al. (mean 17 days).

It is also relevant to discuss the predictive ability of MAS compared to other studies using models that examined different factors regarding their ability to predict discharge destination. Since stroke frequently affects various domains (motor, cognitive, activities of daily living, etc.), it is important to explore the significance of each, in our case, the motor functioning. In a study by Mokler et al. [31], a model based on bladder management, toilet transfer, and memory items from the FIM had an overall predictive value of being discharged home of 66.4% (sensitivity 53.5%, specificity 76.6%), which is lower than the predictability of the MAS in our study. This indicates that overall motor performance, which was not included in the model by Mokler et al., could be an important factor regarding discharge destination. Roberts et al. [32] found that eating, grooming, bowel management, toilet transfer, expression, and memory items of the FIM predicted discharge home by OR 1.06 (95% CI 1.05–1.07) with an AUC of 0.89 (95% CI 0.87–0.92), indicating that it is important to include dysphagia and cognitive ability in the predictive model. Tanwir et al. [33] categorized the FIM motor components and FIM cognitive components into three levels and compared the predictive ability of a high score versus a low score on discharge destination. Regarding the motor components, they found higher odds (OR 4.75, 95% CI 2.14–10.53) while the OR for the cognitive components was 3.02 (95% CI 1.13–8.05). The seemingly big difference in the magnitude of ORs regarding the motor functioning across studies [32, 33] may be due to the categorization of the scale's summed scores by Tanwir et al., which itself may increase the likelihood of missing information and thereby lead to different results [34]. Nevertheless, the findings suggest that combining cognitive and motor functioning scores with other well-known predictors results in a higher predictive ability. It should be noted that in our study, 42% of the patients who obtained a high MAS total score were not discharged home. A possible explanation is thus that their discharge destination was influenced by their cognitive abilities.

Several instruments can be used when trying to determine the future trajectory of the patient, thus offering more flexibility to the clinical practice regarding the choice of instruments. It seems that measuring motor function with a functional scale as the MAS can be applied as a predictive tool in a similar way as a measurement of independence (e.g., the FIM). The advantage of applying independency scales is that severely impaired patients can be included as opposed to MAS, where the patients have to be able to follow verbal instructions. On the other hand, employing the MAS gives indication of which specific motor components are important for returning home (e.g., MAS Item 4 (sitting to standing), or MAS Item 5 (walking) [16]) and can indicate directions for rehabilitation (see Table 4).

Since this study only followed patients during their hospital admittance and not during the complete rehabilitation period, one can ask whether discharge destination is an important outcome in the subacute hospital setting or whether it is only a matter of time to allow for a combination of recovery and rehabilitation to have effect. The need for early prediction of discharge destination arises from studies on early supported discharge to home after stroke. A Cochrane Review found that early supported discharge after stroke decreased the odds for mortality and dependency at follow-up median 6 months after discharge with an OR of 0.80 (95% CI 0.67–0.95) [6]. This might be due to patients being more physically active when discharged home compared to a longer admittance time [35]. In a qualitative study of 27 patients' experiences of early supported discharge, the patients expressed that they perceived home as the first-choice context of rehabilitation [36]. Hence, being discharged home is of importance to the patients at both the physiological and emotional level [35, 36].

We found that scores on the MAS were significant predictors of discharge destination, which adds to the body of knowledge regarding the construct validity of the test. For the rehabilitation department, this could offer some assistance as to administrative planning and clinical goal setting between patient, his/her relatives, and the interdisciplinary team. At the individual patient level, the MAS score might indicate the discharge destination, thus enabling planning of the discharge within the first week of admittance. Research indicates that health care professionals are often challenged regarding predicting discharge destination [37]. Further, it also suggests that there is a need for a better preparation prior to discharge in the transition phase from hospital to home [38]. At the administrative level, the MAS score might provide information on the expected length of stay and discharge destination of the admitted patients and thereby guide assumptions about the number of beds occupied at a certain time point.

The study has some methodological strengths and limitations. Regarding the methodology of validity studies, a priori aims and hypotheses were formulated as recommended by COSMIN [39]. Discharge destination had low risk of information bias since the decision was made by the patient, the patient's relatives, and the interdisciplinary stroke team. The study was prospective with 100% follow-up, and the researchers had no influence on the outcome. Information from the hospital records was based on the unique Danish personal identification number and gave access to information on relevant covariates like age, prestroke housing situation, cohabitant status, and additional treatment. The validity of the hospital records is considered high. There was a low likelihood of selection bias due to demographic and economic factors, since the stroke population was recruited from areas of different sociodemographic settings in the greater Copenhagen.

It could be argued that the MAS was at risk of being exposed to nondifferential misclassification bias, since no prior reliability study was done in this setting, ensuring classification of patients according to their score on the MAS, particularly considering that 10 different physiotherapists were involved in the testing. On the other hand, since previous reliability studies have found the MAS to be highly reliable [13, 17, 21, 23] and a typical sign of nondifferential misclassification bias is the absence of significant results, we believe that the misclassification was unlikely, as even the small sample size of 37 patients yielded significant results in all analyses. The study also has obvious limitations. We did not obtained data on prestroke comorbidity except for motor sequelae after previous stroke. We did not collect data on cognitive disability. The physiotherapists were not blinded to the study protocol, although it is unlikely that this knowledge influenced the outcome. The small sample size also represents a limitation and may impact generalizability of results.

## 5. Conclusion

This prospective cohort study showed that motor function, measured by the MAS, can function as predictor of discharge destination early poststroke in medically stable patients without delirium or severe cognitive disabilities. MAS testing can support the administrative planning of the hospital unit by assisting the discharge planning within short admittance time. It can also assist the physiotherapist and patient with realistic goal setting early poststroke.

## Figures and Tables

**Table 1:** Patient and injury characteristics.

Age, mean (SD)	75.2 (12.2)
Sex, female, no. (%)	17 (45.9)
Prestroke residential status, no. (%)	
Home	33 (89.2)
Municipal unit	0 (0.0)
Nursing home	4 (10.8)
Cohabitant, alone, no. (%)	17 (45.9)
Previous stroke, no. (%)	7 (18.9)
Type of stroke, ischemic, no. (%)	33 (89.2)
Hemisphere, no. (%)	
Left	14 (37.8)
Right	19 (51.4)
Bilateral	4 (10.8)
Additional treatment^a^, no. (%)	30 (81.1)
Hospitalization, days (SD)	18.4 (9.6)
SBP, mmHg (SD)	149.8 (26.6)
DBP, mmHg (SD)	76.9 (11.5)
Pulse (SD)	73.0 (12.7)
BMI (SD)	25.9 (4.7)
FOIS (IQR)	5 (5–7)

*Note:* Mean (SD). Median (IQR). Frequency (%).

Abbreviations: %: percentiles; BMI: body mass index; DBP: diastolic blood pressure; FOIS: Functional Oral Intake Scale; IQR: interquantile range; SBP: systolic blood pressure; SD: standard deviation.

^a^Additional treatment consisted of treatment with thrombolysis, endovascular thrombectomy (EVT), or craniectomy.

**Table 2:** Outcome measures.

MAS, median (IQR)	
Total, 0–48	25.0 (11.5–32.0)
Transfer-mobility, 0–30	16.0 (9.0–22.0)
UE, 0–18	7.0 (2.0–12.5)
Discharge destination (%)	
Home	12 (32.4)
Municipal unit	22 (59.5)
Other hosp/unit	1 (2.7)
Nursing home	2 (5.4)
Deceased	0 (0.0)

*Note:* Median (IQR). Frequency (%).

Abbreviations: %: percentiles; IQR: interquantile range; MAS: Motor Assessment Scale.

**Table 3:** Associations between MAS score and discharge to home.

**Logistic regression, odds ratios (95% CI)**	**Univariate**	**Multivariate**
Total MAS	1.14 (1.04–1.25)	1.30 (1.02–1.65)
Age by MAS		1.00 (1.00–1.00)
Additional treatment		2.36 (0.22–25.56)
Prestroke residential status		1.04 (0.05–22.44)
Cohabitant status		0.42 (0.06–2.95)
Cox regression^a^, hazard ratio (95% CI)		
Total MAS > 23	17.64 (2.23–139.57)	
Transfer-mobility MAS > 15	17.02 (2.15–135.01)	
UE MAS > 6	7.11 (1.54–32.83)	

Abbreviation: CI, confidence interval.

^a^Cox regression of dichotomized MAS and discharge destination.

**Table 4:** Logistic regression and ROC curve of MAS items and discharge to home.

**Item**	**OR (95% CI)**	**R** ^2^	**AUC (95% CI)**
1	2.07 (1.02–4.19)	0.32	0.74 (0.588–0.899)
2	1.81 (1.07–3.05)	0.27	0.73 (0.564–0.889)
3	1.74 (0.99–3.06)	0.15	0.73 (0.570–0.890)
4	2.02 (1.25–3.26)	0.39	0.87 (0.752–0.982)
5	1.39 (0.99–1.96)	0.14	0.68 (0.495–0.868)
6	1.49 (1.04–2.14)	0.19	0.72 (0.551–0.895)
7	1.69 (1.18–2.43)	0.36	0.83 (0.696–0.961)
8	3.31 (1.27–8.63)	0.34	0.78 (0.612–0.938)

Abbreviations: AUC, area under curve; CI, confidence interval; OR, odds ratio; *R*^2^, *R*-squared.

## Data Availability

The access to the clinical data used to support the findings of this study is restricted by the Danish Data Protection Agency, journal number 05307, and the National Committee on Health Research Ethics, Capital Region of Denmark, journal number 17002843, in order to protect the patient privacy.
